# Effect of Severe Fever With Thrombocytopenia Syndrome Virus Genotype on Disease Severity, Viral Load, and Cytokines in South Korea

**DOI:** 10.1093/ofid/ofae508

**Published:** 2024-09-20

**Authors:** Ji-Soo Kwon, Ji Yeun Kim, Choi Young Jang, Ju Yeon Son, Woori Kim, Taeeun Kim, Se Yoon Park, Min-Chul Kim, Seong Yeon Park, Hye Hee Cha, Hyeon Mu Jang, Min-Jae Kim, Yong Pil Chong, Sang-Oh Lee, Sang-Ho Choi, Yang Soo Kim, Sung-Han Kim

**Affiliations:** Department of Infectious Diseases, Asan Medical Center, University of Ulsan College of Medicine, Seoul, Republic of Korea; Department of Infectious Diseases, Asan Medical Center, University of Ulsan College of Medicine, Seoul, Republic of Korea; Department of Infectious Diseases, Asan Medical Center, University of Ulsan College of Medicine, Seoul, Republic of Korea; Department of Infectious Diseases, Asan Medical Center, University of Ulsan College of Medicine, Seoul, Republic of Korea; Department of Infectious Diseases, Asan Medical Center, University of Ulsan College of Medicine, Seoul, Republic of Korea; Division of Infectious Diseases, Gyeongsang National University Hospital, Gyeongsang National University School of Medicine, Jinju, Republic of Korea; Division of Infectious Diseases, Department of Internal Medicine, Soonchunhyang University Seoul Hospital, Soonchunhyang University College of Medicine, Seoul, Republic of Korea; Division of Infectious Diseases, Chung-Ang University Hospital, Seoul, Republic of Korea; Department of Infectious Diseases, Dongguk University Ilsan Hospital, Goyang, Republic of Korea; Department of Infectious Diseases, Asan Medical Center, University of Ulsan College of Medicine, Seoul, Republic of Korea; Department of Infectious Diseases, Asan Medical Center, University of Ulsan College of Medicine, Seoul, Republic of Korea; Department of Infectious Diseases, Asan Medical Center, University of Ulsan College of Medicine, Seoul, Republic of Korea; Department of Infectious Diseases, Asan Medical Center, University of Ulsan College of Medicine, Seoul, Republic of Korea; Department of Infectious Diseases, Asan Medical Center, University of Ulsan College of Medicine, Seoul, Republic of Korea; Department of Infectious Diseases, Asan Medical Center, University of Ulsan College of Medicine, Seoul, Republic of Korea; Department of Infectious Diseases, Asan Medical Center, University of Ulsan College of Medicine, Seoul, Republic of Korea; Department of Infectious Diseases, Asan Medical Center, University of Ulsan College of Medicine, Seoul, Republic of Korea

**Keywords:** cytokines, genotype, severe fever with thrombocytopenia syndrome, SFTS, viral load

## Abstract

**Background:**

Severe fever with thrombocytopenia syndrome (SFTS) is an emerging tick-borne disease caused by *Bandavirus dabieense* (SFTS virus [SFTSV]). Recently, at least 6 different genotypes of SFTSV have been identified, with genotypes A, D, and F dominant in China and B dominant in Japan and Korea. This study investigated the effect of SFTSV genotypes circulating in South Korea on disease severity, viral load, and cytokine profile.

**Methods:**

We prospectively enrolled 70 patients with SFTS from July 2015 to June 2022. Serial plasma samples were obtained during hospitalization and analyzed. Viral load was measured by real-time reverse-transcription polymerase chain reaction. Partial sequences of the viral genome were analyzed for genotyping. Plasma concentrations of 17 cytokines were measured by multiplex-bead immunoassay.

**Results:**

Of 70 samples, 51 could be genotyped. Genotype B was predominant (80.4%) and other genotypes were uncommon. Intensive care unit admission rates (51.2% vs 50.0%) and mortality rates (26.8% vs 40.0%) did not show any significant differences between genotype B and non-B genotypes. The initial viral load did not show any significant differences (3.59 vs 3.64 log copies/μL), whereas viral load measured at hospital day 3–4 tended to be higher in genotype B than non-B genotypes (3.83 vs 1.83 log copies/μL, *P* = .07). Additionally, the plasma concentrations of interferon-α, interleukin 10, and interferon-γ–induced protein 10, which are closely related to mortality in cases of SFTS, did not show any significant differences.

**Conclusions:**

SFTSV genotype B was the prevalent genotype in South Korea, with no genotype-specific difference in clinical outcomes, initial viral load, or cytokine profiles.

## Teaser

We analyzed genotypes of severe fever with thrombocytopenia syndrome (SFTS) virus circulating in South Korea. The majority of analyzed cases was classified as genotype B. There were no genotype-specific differences in factors associated with fatal outcome of SFTS.

Severe fever with thrombocytopenia syndrome (SFTS) is an emerging tick-borne infectious disease caused by *Bandavirus dabieense* (severe fever with thrombocytopenia syndrome virus [SFTSV]) in the family Phenuiviridae in East Asia. The genome of SFTSV has negative-stranded RNA segments including segment L (large), M (middle), and S (small). Similar to many other RNA viruses, SFTSV undergoes several mutations during proliferation within the host and recombination during transmission. Recently, at least 6 different genotypes of SFTSV have been identified, with genotypes A, D, and F dominant in China and B dominant in Japan and South Korea [1, 2]. Previous studies have described the lethality of the predominant genotypes in each region [3, 4]; interestingly, the mortality rate of SFTS in China was relatively low at 6.2%–18.5% [5, 6], compared to the higher rates of 18.7%–35.1% [7, 8] in South Korea and Japan. A previous study that confirmed the pathogenicity of SFTSV using an animal model indicated that genotype B may lead to higher mortality than non-B genotypes, suggesting that genotype may play a crucial role in the pathogenesis of SFTS [3]. However, the impact of the SFTSV genotype on the course of the disease and immune responses in patients is limited. In the present study, we investigated the effect of SFTSV genotypes circulating in South Korea on disease severity, viral load, and cytokine profile.

## MATERIALS AND METHODS

### Study Population and Sample Collection

We prospectively enrolled confirmed patients with SFTS admitted to 5 university-affiliated hospitals in South Korea (Asan Medical Center, Gyeongsang National University Hospital, Soonchunhyang University Seoul Hospital, Chung-Ang University Hospital, and Dongguk University Ilsan Hospital), from July 2015 to June 2022. Patients nationwide except Jeju island were enrolled, depending on the regional location of each participating hospital and its role as a tertiary hospital (Asan Medical Center, Chung-Ang University Hospital, and Gyeongsang University Hospital). SFTS was confirmed by the detection of viral RNA with real-time reverse-transcription polymerase chain reaction (RT-PCR), as previously described [9]. Infectious disease physicians at the participating hospitals followed the enrolled patients from admission to discharge. The treatments, including blood products, intravenous fluid, and other supportive treatments, were provided at the discretion of the attending infectious disease specialists. They reviewed the electronic medical records of the enrolled patients, including clinical and laboratory data. For analysis, peripheral blood samples were serially collected in ethylenediaminetetraacetic acid vacutainer tubes from patients who agreed with serial sampling until the hospital discharge. Then the vacutainer containing whole blood was centrifuged at 2500 RPM and the separated plasma was then stored at −80°C until analysis, including genotyping, viral load, and cytokine measurement. All individuals were informed of the nature of the study and written informed consent was obtained from them prior to enrollment. The study protocol was approved by the institutional review board (IRB) committee of each participating hospital (Asan Medical Center IRB number 2016-0748, Gyeongsang National University Hospital IRB number 2019-10-019, Soonchunhyang University Seoul Hospital IRB number 2016-09-001, Chung-Ang University Hospital IRB number 2016-033-468, Dongguk University Ilsan Hospital IRB number 2016-01-088).

### Viral Load Measurement

The viral load of patients with SFTS was measured by 1-step multiplex real-time RT-PCR, as previously described [9]. In brief, viral RNA was extracted from plasma samples using the Qiagen RNeasy Mini Kit (Qiagen, Hilden, Germany). To quantify viral load, we detected the segment S and M of SFTSV genome, and human β-actin gene was used as an internal control. The primers and probe used in this study are shown in Supplementary Table 1. The reaction mixture was prepared with LightCycler Multiplex RNA Virus Master (Roche Diagnostics, Indianapolis, Indiana) and RT-PCR was conducted using the LightCycler 96 system (Roche Diagnostics) as follows: reverse transcription at 50°C for 10 minutes, preincubation at 95°C for 10 minutes, followed by 45 cycles of 2-step amplification (95°C for 5 seconds and 56°C for 30 seconds). The SFTSV RNA copy number was determined based on a standard curve generated from the cycle threshold values of in vitro transcript RNA. The detection limit of real-time RT-PCR was 4.3 copies/μL of plasma sample.

### Genotyping

After viral load measurement, additional RNA was extracted from the remaining plasma samples for genotypic classification. The partial sequences of segment S and M of the SFTSV genome were analyzed. In brief, we extracted viral RNA from the initially obtained specimen of each participant. The viral RNA was subjected to 1-step conventional RT-PCR using AccuPower RocketScript RT-PCR PreMix (Bioneer, Daejeon, South Korea) with designated primers under specific PCR conditions as previously published by the Korea Disease Control and Prevention Agency [10, 11]: reverse transcription at 50°C for 30 minutes, denaturation at 95°C for 15 minutes, followed by 35 cycles of 3-step amplification (95°C for 20 seconds, 58°C [for segment M] or 55°C [for segment S] for 40 seconds, and 72°C for 30 seconds), and final extension at 72°C for 5 minutes. The primers used in this study are shown in Supplementary Table 1. The amplified DNA underwent gel electrophoresis for band size confirmation (segment S: 567 bp, segment M: 560 bp), followed by elution and cloning using the TA cloning method with the T-Blunt PCR Cloning kit (SolGent, Daejeon, South Korea). Using the maximum likelihood method, we constructed phylogenetic trees for segment S and M incorporating sequences obtained in this study along with 512 sequences analyzed in previous studies [3, 4, 12].

### Cytokine Measurement

We simultaneously measured the following 16 selected cytokines in the plasma samples using a cytometric bead array (BD Biosciences, San Jose, California): interferon alpha (IFN-α), interferon gamma (IFN-γ), interleukin (IL)-1β, IL-2, IL-6, IL-8, IL-10, IL-12p40, IL-13, IL-17A, IFN-γ–induced protein 10 (IP-10), tumor necrosis factor alpha (TNF-α), monocyte chemotactic protein 1 (MCP-1), macrophage inflammatory protein 1α (MIP-1α), regulated on activation and normally T-cell expressed and secreted (RANTES), granulocyte colony-stimulating factor (G-CSF), granulocyte-macrophage colony-stimulating factor, and vascular endothelial growth factor. Capture beads coated with antibodies against each cytokine and fluorophore-conjugated detection antibodies were used to detect the proteins. Data were acquired using the FACS CANTO Ⅱ flow cytometer, FACS Diva software (BD Biosciences), and FlowJo software (FlowJo LLC, Ashland, Oregon).

### Statistical Analysis

For statistical analyses, categorical variables were compared by Fisher exact test or χ^2^ test, and continuous variables were compared by unpaired *t* test, Mann-Whitney *U* test, or Kruskal-Wallis test. All tests of significance were 2-tailed, and *P* values <.05 were considered statistically significant. Statistical analyses were performed using GraphPad Prism 9.1.2 (GraphPad Software, La Jolla, California).

## RESULTS

A total of 70 patients with confirmed SFTS were enrolled in the present study. Of 70 enrolled patients, 19 patients had a low viral load that was insufficient for genotyping. Consequently, a total of 51 samples from 51 patients were used for genotyping analysis (Supplementary Figure 1), which identified 5 different genotypes of SFTSV (Supplementary Table 2). Among the 51 samples examined, genotype B (including subtypes B-1, B-2, and B-3) was the predominant genotype, accounting for 41 samples (80.4%). Other genotypes included genotype A in 3 samples (5.9%), genotype D in 1 sample (2.0%), genotype E in 1 sample (2.0%), genotype F in 2 samples (3.9%), and reassorted genotypes in 3 samples (5.9%). The median age, male sex, clinical characteristics, and past medical histories were not different between patients with genotype B and those with non-B genotypes (Table 1). Based on laboratory findings, patients infected with genotype B had significantly lower levels of white blood cells (median, 1605 [interquartile range {IQR}, 1100–2200] vs 2350 [IQR, 1650–6500] cells/μL; *P* = .01) and tended to have lower levels of alkaline phosphatase (median, 69.0 [IQR, 59.0–100.0] vs 132.0 [IQR, 65.0–241.0] IU/L; *P* = .09) compared with patients with non-B genotypes.

**Table 1.** Baseline Characteristics of Patients Infected With Genotype B and Non-B Genotypes of Severe Fever With Thrombocytopenia Syndrome Virus

Characteristic	Total (n = 51)	Genotype B (n = 41)	Non-B Genotypes^a^ (n = 10)	*P* Value
Age, y, median (IQR)	65.0 (58.0−72.0)	65.0 (59.5−72.0)	64.0 (53.0–71.0)	.62
Male sex	31 (60.8)	24 (58.5)	7 (70.0)	.72
Clinical characteristics				
Fever (≥38°C)	48 (94.1)	39 (95.1)	9 (90.0)	.49
Skin rash	6 (11.8)	5 (12.2)	1 (10.0)	.99
Bleeding^b^	8 (15.7)	8 (19.5)	0	.33
Myalgia	28 (54.9)	24 (58.6)	4 (40.0)	.32
General weakness	35 (68.6)	29 (70.7)	6 (60.0)	.71
Stomachache	15 (29.4)	12 (29.3)	3 (30.0)	.99
Diarrhea	20 (39.2)	15 (36.6)	5 (50.0)	.49
Altered mental status	30 (58.8)	24 (58.5)	6 (60.0)	.99
Concomitant *Aspergillus* infection^c^	11 (21.6)	9 (22.0)	2 (20.0)	.99
Underlying diseases				
Chronic lung diseases^d^	4 (7.8)	3 (7.3)	1 (10.0)	.99
Diabetes mellitus	13 (25.5)	12 (29.3)	1 (10.0)	.42
Chronic liver diseases^e^	4 (7.8)	4 (9.8)	0	.57
Autoimmune diseases^f^	1 (2.0)	0	1 (10.0)	.20
Solid tumor^g^	4 (7.8)	3 (7.2)	1 (10.0)	.99
Hematologic malignancy^h^	2 (3.9)	1 (2.4)	1 (10.0)	.36
Laboratory findings at admission, median (IQR)				
WBC count, cells/μL	1800 (1200–2425)	1605 (1100–2200)	2350 (1650–6500)	.01
Hemoglobin, g/dL	14.0 (12.8–15.0)	14.0 (12.7–15.0)	14.0 (12.9–14.5)	.74
Platelet, ×10^3^/μL	58.0 (42.5–78.5)	60.5 (40.5–81.3)	53.0 (44.0–65.5)	.66
BUN, mg/dL	15.1 (11.4–22.0)	14.2 (11.0–23.0)	16.9 (11.5–21.4)	.81
Creatinine, mg/dL	0.81 (0.7–1.1)	0.8 (0.7–1.1)	0.9 (0.8–1.3)	.25
AST, IU/L	246.0 (94.0–579.5)	249.0 (93.5–607.0)	246.0 (142.0–579.5)	.92
ALT, IU/L	96.0 (59.0–198.0)	95.0 (54.5–201.3)	96.0 (53.5–135.5)	.52
ALP, IU/L	72.0 (60.0–129.5)	69.0 (59.0–100.0)	132.0 (65.0–241.0)	.09
CRP, mg/dL	0.5 (0.2–1.0)	0.4 (0.2–1.0)	0.6 (0.5–2.8)	.20
Time from hospital admission to hospital discharge or death, d, median (IQR)	9.0 (6.0–12.8)	9.5 (6.0–13.8)	8.5 (4.5–9.8)	.15
Clinical courses				
ICU admission	26 (51.0)	21 (51.2)	5 (50.0)	.99
CNS involvement^i^	28 (54.9)	22 (53.7)	6 (60.0)	.99
Lung involvement^j^	20 (39.2)	16 (39.0)	4 (40.0)	.99
Renal dysfunction^k^	17 (33.3)	12 (29.3)	5 (50.0)	.27
In-hospital mortality	15 (29.4)	11 (26.8)	4 (40.0)	.45
Treatments				
Ribavirin	18 (35.3)	13 (31.7)	5 (50.0)	.30
Doxycycline	37 (72.5)	30 (73.2)	7 (70.0)	.99
Plasmapheresis	34 (66.7)	29 (70.7)	5 (50.0)	.27
Convalescent plasma therapy	10 (19.6)	8 (19.5)	2 (20.0)	.99

Data are presented as No. (%) unless otherwise indicated.

Abbreviations: ALT, alanine aminotransferase; ALP, alkaline phosphatase; AST, aspartate aminotransferase; BUN, blood urea nitrogen; CNS, central nervous system; CRP, C-reactive protein; ICU, intensive care unit; IQR, interquartile range; WBC, white blood cell.

^a^All genotypes except genotype B.

^b^Bleeding was defined as hemorrhagic signs such as epistaxis, hemoptysis, hematemesis, melena, hematochezia, and macroscopic hematuria.

^c^Concomitant *Aspergillus* infection was defined as coinfection of putative aspergillosis based on clinical, radiological, and mycological criteria by the *Asp*ICU algorithm.

^d^Chronic lung diseases were defined as chronic obstructive pulmonary diseases and restrictive lung diseases.

^e^Chronic liver diseases were defined as chronic hepatitis B virus or hepatitis C virus infection and liver cirrhosis due to various causes.

^f^Autoimmune diseases were defined as rheumatologic diseases including lupus, dermatomyositis, and rheumatoid arthritis; inflammatory bowel diseases such as ulcerative colitis and Crohn disease; and other autoimmune endocrine or skin diseases.

^g^Solid tumor was defined as carcinoma or sarcoma.

^h^Hematologic malignancy was defined as lymphoma, multiple myeloma, and leukemia.

^i^CNS involvement referred to cases when meningoencephalitis or encephalopathy occurs due to severe fever with thrombocytopenia syndrome.

^j^Lung involvement referred to cases when the patient had abnormal chest X-ray infiltration that could not be explained by other causes.

^k^Renal dysfunction referred to cases when the patient requires continuous renal replacement therapy, due to acute renal failure.

**Figure 1.**
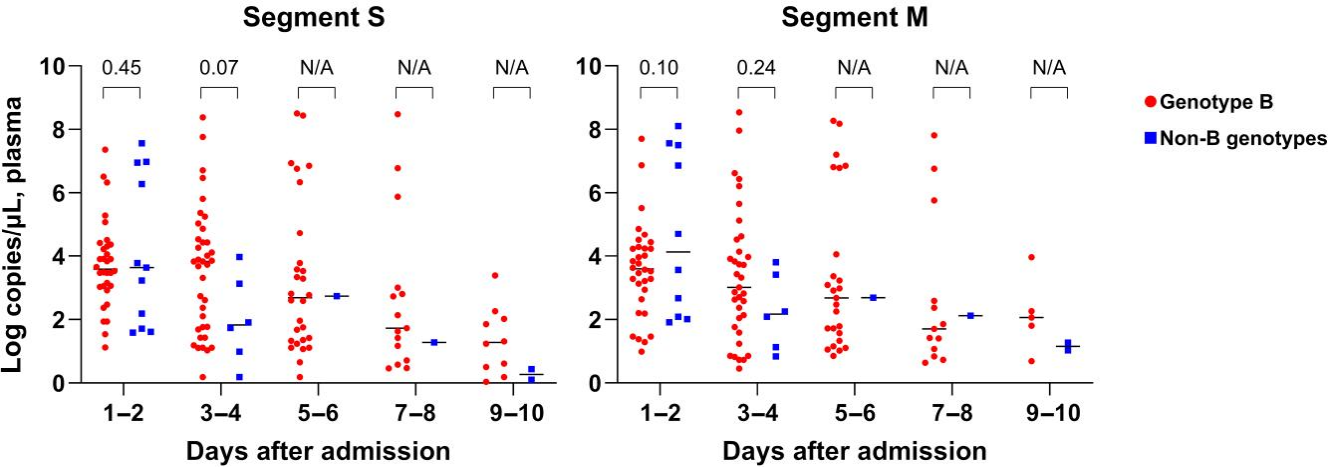
Viral load kinetics of patients infected with severe fever with thrombocytopenia syndrome virus genotype B and non-B genotypes. The values of each group were compared by Mann-Whitney *U* test. Abbreviation: N/A, not available.

The detailed viral load kinetics of patients with SFTS after admission are shown in Figure 1. The viral loads of both genotype B and non-B genotypes were decreased over time. Patients infected with non-B genotypes had a modestly higher titer of segment M in the initial viral load (median, 3.60 [IQR, 2.72–4.28] vs 4.13 [IQR, 2.07–7.51] log copies/μL plasma; *P* = .10), whereas patients infected with genotype B tended to exhibit a slightly elevated segment S titer on day 3–4 of hospitalization (median, 3.83 [IQR, 1.73–4.70] vs 1.83 [IQR, 0.79–3.34] log copies/μL plasma; *P* = .07).

**Figure 2.**
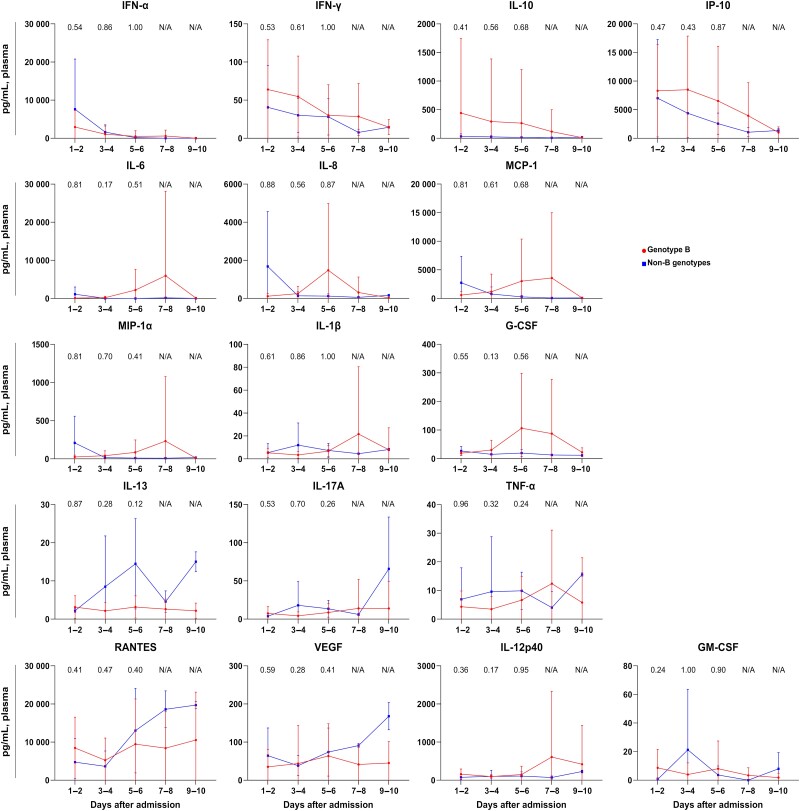
Detailed cytokine kinetics of patients infected with severe fever with thrombocytopenia syndrome virus genotype B and non-B genotypes. The values of each group were compared by Mann-Whitney *U* test. In the period between hospital day 7 and 10, the sample size was insufficient for analysis; therefore, the results are expressed as not available. Abbreviations: G-CSF, granulocyte colony-stimulating factor; GM-CSF, granulocyte-macrophage colony-stimulating factor; IFN-α, interferon alpha; IFN-γ, interferon gamma; IL, interleukin; IP-10, interferon gamma–induced protein 10; MCP-1, monocyte chemotactic protein 1; MIP-1α, macrophage inflammatory protein 1α; N/A, not available; RANTES, regulated on activation and normally T-cell expressed and secreted; TNF-α, tumor necrosis factor alpha; VEGF, vascular endothelial growth factor.

A total of 101 plasma specimens were available for multiplex cytokine bead array. Among the 17 cytokines analyzed in this study, the plasma concentrations of IFN-α, IFN-γ, IL-10, IP-10, IL-6, IL-8, MCP-1, MIP-1a, IL-1b, and G-CSF, which have been associated with mortality in cases of SFTS [13], did not exhibit any significant differences between genotype B and non-B genotypes (Figure 2).

**Figure 3.**
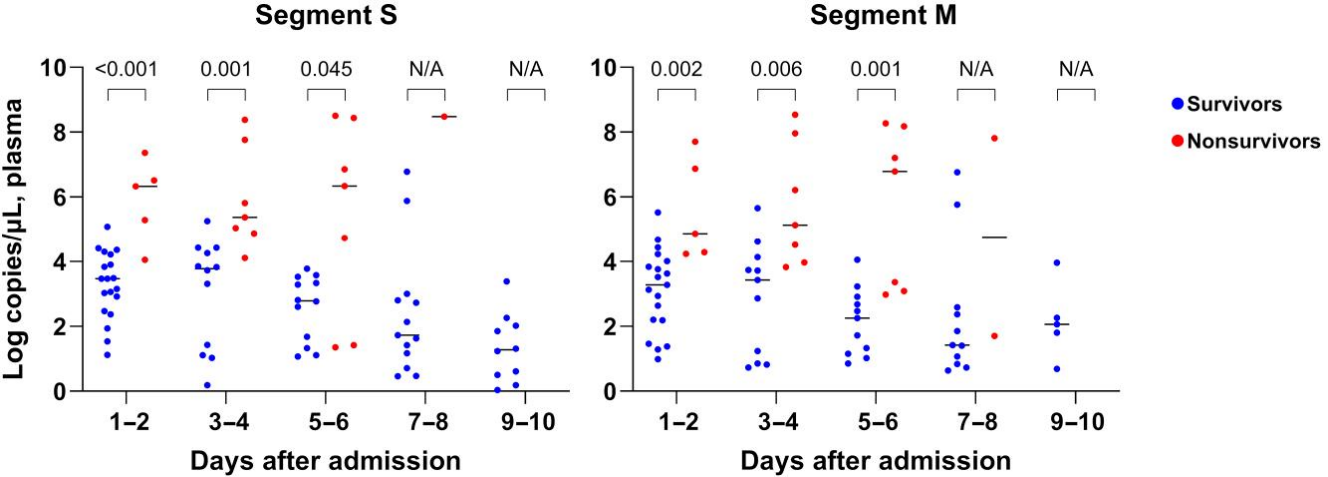
Viral load kinetics of survivors and nonsurvivors infected with severe fever with thrombocytopenia syndrome virus genotype B. The values of each group were compared by Mann-Whitney *U* test. Abbreviation: N/A, not available.

**Figure 4.**
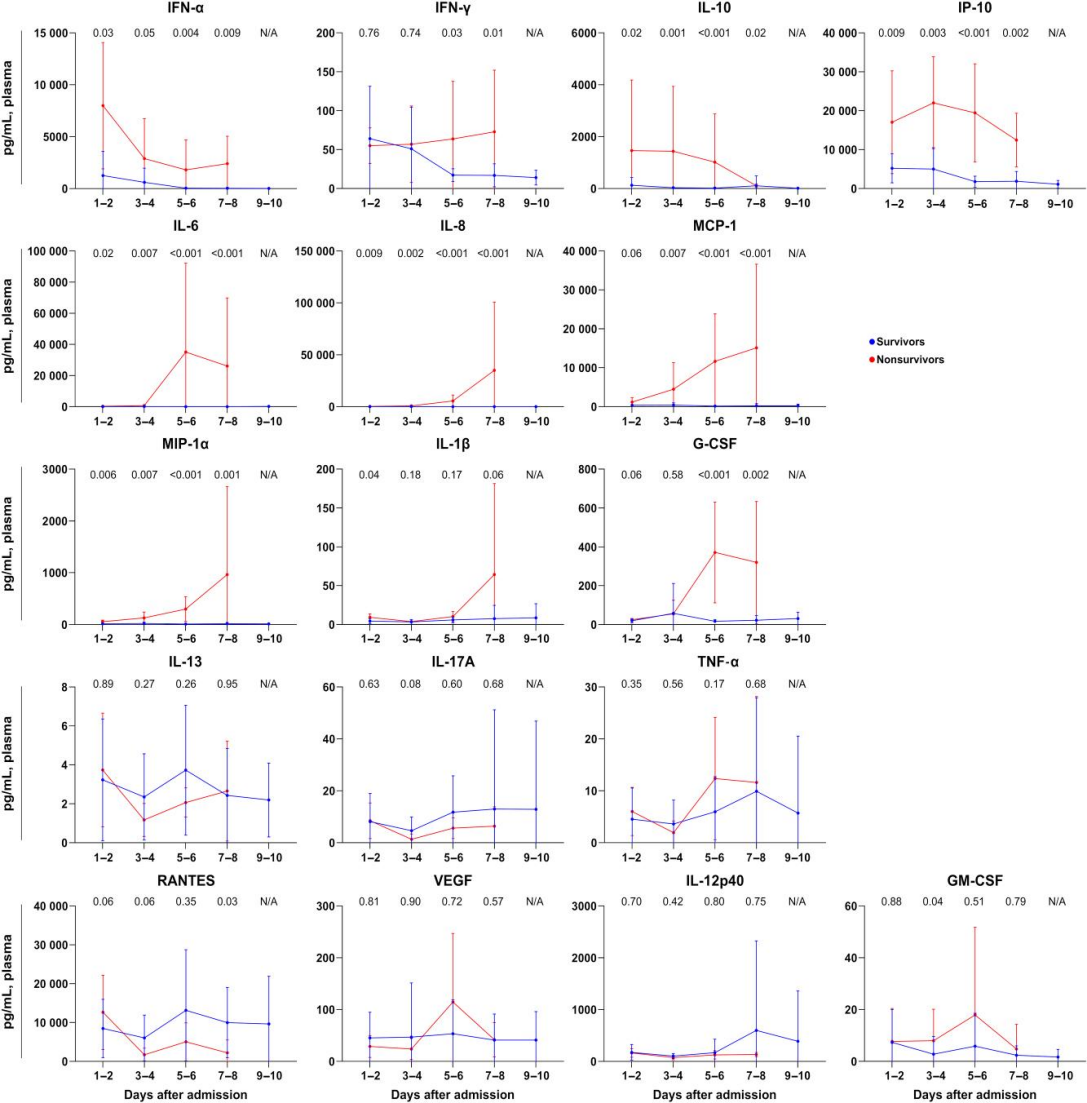
Detailed cytokine kinetics of survivors and nonsurvivors infected with severe fever with thrombocytopenia syndrome virus genotype B. The values of each group were compared by Mann-Whitney *U* test. Abbreviations: G-CSF, granulocyte colony-stimulating factor; GM-CSF, granulocyte-macrophage colony-stimulating factor; IFN-α, interferon alpha; IFN-γ, interferon gamma; IL, interleukin; IP-10, interferon gamma–induced protein 10; MCP-1, monocyte chemotactic protein 1; MIP-1α, macrophage inflammatory protein 1α; N/A, not available; RANTES, regulated on activation and normally T-cell expressed and secreted; TNF-α, tumor necrosis factor alpha; VEGF, vascular endothelial growth factor.

As the majority of the samples were identified as genotype B, we examined the viral load and cytokine kinetics for both survivors and nonsurvivors among patients infected with genotype B. In comparison with survivors, nonsurvivors consistently exhibited significantly higher viral loads in the first week of hospitalization (Figure 3). Plasma levels of IFN-α, IL-10, IP-10, IL-6, IL-8, MIP-1α, and MCP-1 remained elevated in nonsurvivors throughout the admission period (Figure 4). IFN-α, IL-10, and IP-10 exhibited a decreasing pattern, whereas IL-6, IL-8, MIP-1α, and MCP-1 showed an increasing trend. G-CSF and IFN-γ levels showed a gradual increase from the first week of admission, with markedly higher levels observed in deceased patients.

## DISCUSSION

The importance of SFTSV genotypes has emerged recently; their significant genetic diversity is caused by frequent reassortment events and rapid evolutions in various hosts [14–17]. Phylogenetic analysis of the virus has identified 6 main genotypes with further subgenotyping within groups. The prevalence of these genotypes varies by geographic regions and may influence differences in case fatality rates across East Asia [3]. However, there have been limited reports on this aspect. In this study, we examined how different SFTSV genotypes found in South Korea affect the severity of disease, viral burden, and cytokine expressions. Previous studies have also reported that genotype B is the predominant genotype of SFTSV in South Korea among humans, ticks, and animals [2, 3, 18, 19]. Most patients included in our study were diagnosed following outdoor activities nationwide, excluding Jeju island, and the majority of the analyzed SFTSV sequences were identified as genotype B.

To the best of our knowledge, a previous study of Yun et al [3] is the only publication confirming fatal outcomes related to the SFTSV genotype in South Korea, and no other studies have investigated clinical outcomes besides mortality. They analyzed 116 SFTSV isolates and reported that genotype B strains were the most prevalent and associated with the highest case fatality rate [3]. In this study, baseline characteristics, clinical outcomes, and laboratory findings were compared according to the SFTSV genotype identified from patient samples. There were no significant differences between genotype B and non-B genotypes in symptoms, clinical outcomes, or underlying diseases; however, patients infected with genotype B exhibited lower white blood cell counts and alkaline phosphatase levels. Furthermore, no significant differences were observed when genotype B and non-B genotypes were compared in terms of viral load and cytokine levels. The comparable age and intensive care unit admission rate can explain the similar mortality rate in the current study. Given these factors are known to be significant factors influencing mortality, it is reasonable to observe similar mortality rates in both groups. While genotype can play a role in clinical courses, it is important to consider host factors that might also influence mortality [20]. In our study, the distribution of ages and comorbidities was similar between groups, which could further explain the comparable mortality rates. Further research exploring genotypes and their interactions with clinical variables would be meaningful to fully understand their impact on clinical courses.

In a previous study by Dai et al, compared with other genotypes, genotype D was associated with the highest case fatality rate, with significantly elevated virus titers and cytokine concentrations [4]. Due to the insufficient number of genotype D cases in the present study, a comparative analysis with other genotypes could not be performed. Nevertheless, among patients infected with SFTSV genotype B, which was identified as the predominant genotype in South Korea, higher viral titers and cytokine levels were observed in nonsurvivors compared with survivors. This finding is consistent with that of our previous study [13] as some patients overlapped between the studies, with most of them belonging to the genotype B group. However, different predominant genotypes might partially contribute to discrepancies in case fatality rates across countries. The geographical distribution of the SFTSV genotype has been reported [1], and cumulative mortality rates in different regions are as follows: South Korea (genotype B), 18.7% (2013–2022) [5]; Japan (genotype B), 35.1% (2013–2021) [6]; Jiangsu province, China (genotypes A and F), 6.2% (2011–2022) [7]; and Shandong province, China (genotypes D–F), 18.5% (2011–2019) [8]. Nevertheless, besides the SFTSV genotype, the patients’ individual characteristics might influence survival after infection.

Our study has several limitations. First, a meaningful analysis of genotypes other than B was challenging due to the limited number of patients and specimens for these genotypes. For a more comprehensive analysis, additional studies with a higher number of subjects according to the region and genotype are necessary. Second, neither antibody nor cell-mediated immune responses according to the genotype were measured. A previous study showed higher antibody responses for a specific genotype of SFTSV compared with other genotypes, suggesting the possibility of immune evasion [4]. Therefore, further studies on adaptive immune responses and SFTSV genotypes are needed for disease prevention, treatment, and vaccine development.

In conclusion, SFTSV genotype B was the predominant genotype in South Korea, and there was no genotype-specific difference in clinical outcomes, initial viral load, or cytokine kinetics. The limited number of patients for genotypes other than B highlights the need for additional studies with an expanded number of subjects for each genotype.

## Supplementary Data

Supplementary materials are available at *Open Forum Infectious Diseases* online. Consisting of data provided by the authors to benefit the reader, the posted materials are not copyedited and are the sole responsibility of the authors, so questions or comments should be addressed to the corresponding author.

## Supplementary Material

ofae508_Supplementary_Data

## Notes

***Author contributions.*** All authors contributed to the study conception and resource collection. J.-S. K. performed data collection and analysis and wrote the first draft of the manuscript. All authors read and made intellectual contributions to versions of the manuscript and approved the final version of the manuscript.

***Financial support*.** This study was supported by grants from the Bio&Medical Technology Development Program of the National Research Foundation (NRF) funded by the Korean government (MSIT) (grant number RS-2023-00219002), and the National Institute of Health, Republic of Korea (grant number 2020-ER5304-02).
