# Supplementary material for: Effect of Severe Fever With Thrombocytopenia Syndrome Virus Genotype on Disease Severity, Viral Load, and Cytokines in South Korea
Source: Open Forum Infect Dis. 2024 Sep 20;11(9):ofae508. doi: 10.1093/ofid/ofae508 (PMC11414404; doi:10.1093/ofid/ofae508)

**Supplementary Table 1. The primers and probes used in this study.**

| **Assay** | **Name** | **Start** | **Sequence (5' to 3')** | **Modification** |
| --- | --- | --- | --- | --- |
| **Viral load measurement** | SFTS SF (NC_043451) | 1453 | CGAGAGAGCTGGCCTATGAA |  |
| (Real-time RT-PCR [9]) | SFTS SR | 1601 | TTCCCTGATGCCTTGACGAT |  |
|  | SFTS SP | 1553 | TGTCTTTGCCCTGACTCGAGGCA | 5' FAM, 3' BHQ1 |
|  | SFTS MF (NC_043452) | 316 | ATGCTTGTCGTGAAGAAGGC |  |
|  | SFTS MR | 446 | CTAGACTTCCCACTGCCACA |  |
|  | SFTS MP | 400 | ACTTTTGATGGATACGTAGGCTGGGGC | 5' Cy5, 3' BHQ2 |
|  | ACTB F (NC000007.14) | 1670 | ACTAACACTGGCTCGTGTGA |  |
|  | ACTB R | 1774 | CTTGGGATGGGGAGTCTGTT |  |
|  | ACTB P | 1700 | AGGCTGGTGTAAAGCGGCCTTGG | 5' HEX, 3' BHQ1 |
| **Genotyping** | SF3 (NC_043451) | 1075 | GGGTCCCTGAAGGAGTTGTAAA |  |
| (Conventional RT-PCR [10,11]) | SR1 | 1642 | TGGTGAGCAGCAGCTCAATT |  |
|  | MF3 (NC_043452) | 1690 | GATGAGATGGTCCATGCTGATTCT |  |
|  | MR2 | 2249 | CTCATGGGGTGGAATGTCCTCAC |  |

**Supplementary Table 2. Information on the SFTSV sequences analyzed in this study.**

| **No** | **Strain** | **Genotype** | **Geographical origin** | **Year** | **Accession number** | | **Sequence source** |
| --- | --- | --- | --- | --- | --- | --- | --- |
|  |  |  |  |  | **Segment M** | **Segment S** |  |
| 1 | K15-01 | B-3 | Chungcheong | 2015.07 | PQ122705 | PQ122750 | GenBank |
| 2 | K16-01 | B-1 | Seoul | 2016.07 | PQ122706 | PQ122751 | GenBank |
| 3 | K16-02 | B-2 | Gyeongsang | 2016.07 | PQ122707 | PQ122752 | GenBank |
| 4 | K16-03 | B-3 | Gyeongsang | 2016.09 | PQ122708 | PQ122753 | GenBank |
| 5 | K16-04 | B-1 | Gyeonggi | 2016.09 | PQ122709 | PQ122754 | GenBank |
| 6 | K16-05 | B-1 | Gyeonggi | 2016.09 | PQ122710 | PQ122755 | GenBank |
| 7 | K16-06 | B-2 | Gyeongsang | 2016.09 | PQ122711 | N/A | GenBank |
| 8 | K16-07 | F | Seoul | 2016.09 | PQ122712 | PQ122756 | GenBank |
| 9 | K16-08 | B-3 | Chungcheong | 2016.10 | PQ122713 | N/A | GenBank |
| 10 | K16-09 | Reassorted | Gangwon | 2016.10 | PQ122714 | N/A | GenBank |
| 11 | K16-10 | A | Gangwon | 2016.10 | PQ122715 | PQ122757 | GenBank |
| 12 | K16-11 | A | Jeolla | 2016.10 | N/A | PQ122758 | GenBank |
| 13 | K16-12 | B-2 | Chungcheong | 2016.10 | PQ122716 | PQ122759 | GenBank |
| 14 | K17-01 | F | Gyeonggi | 2017.06 | PQ122717 | PQ122760 | GenBank |
| 15 | K17-02 | B-3 | Gyeongsang | 2017.07 | PQ122718 | PQ122761 | GenBank |
| 16 | K17-03 | B-2 | Gyeongsang | 2017.07 | PQ122719 | PQ122762 | GenBank |
| 17 | K17-04 | B-2 | Gyeongsang | 2017.08 | PQ122720 | N/A | GenBank |
| 18 | K17-05 | B-1 | Gyeonggi | 2017.09 | PQ122721 | N/A | GenBank |
| 19 | K17-06 | B-1 | Gyeonggi | 2017.10 | PQ122722 | N/A | GenBank |
| 20 | K17-07 | B-1 | Gyeonggi | 2017.10 | PQ122723 | N/A | GenBank |
| 21 | K17-08 | B-2 | Seoul | 2017.10 | PQ122724 | N/A | GenBank |
| 22 | K18-01 | B-2 | Gyeonggi | 2018.05 | PQ122725 | N/A | GenBank |
| 23 | K18-02 | B-2 | Gyeongsang | 2018.05 | PQ122726 | N/A | GenBank |
| 24 | K18-03 | B-1 | Gangwon | 2018.06 | PQ122727 | N/A | GenBank |
| 25 | K18-04 | B-2 | Chungcheong | 2018.06 | PQ122728 | N/A | GenBank |
| 26 | K18-05 | B-3 | Gangwon | 2018.06 | PQ122729 | N/A | GenBank |
| 27 | K18-06 | B-2 | Gyeongsang | 2018.06 | PQ122730 | PQ122763 | GenBank |
| 28 | K18-07 | B-2 | Gyeongsang | 2018.08 | PQ122731 | PQ122764 | GenBank |
| 29 | K18-08 | Reassorted | Gangwon | 2018.09 | PQ122732 | PQ122765 | GenBank |
| 30 | K18-09 | D | Jeolla | 2018.10 | PQ122733 | PQ122766 | GenBank |
| 31 | K18-10 | B-1 | Gyeonggi | 2018.10 | N/A | PQ122767 | GenBank |
| 32 | K19-01 | B-1 | Chungcheong | 2019.08 | PQ122734 | PQ122768 | GenBank |
| 33 | K19-02 | B-1 | Seoul | 2019.09 | PQ122735 | PQ122769 | GenBank |
| 34 | K19-03 | B-1 | Gyeonggi | 2019.10 | N/A | PQ122770 | GenBank |
| 35 | K19-04 | B-3 | Gyeongsang | 2019.10 | PQ122736 | PQ122771 | GenBank |
| 36 | K19-05 | B-2 | Gyeonggi | 2019.10 | PQ122737 | N/A | GenBank |
| 37 | K19-06 | B-2 | Gangwon | 2019.10 | PQ122738 | N/A | GenBank |
| 38 | K20-01 | B-1 | Chungcheong | 2020.06 | PQ122739 | PQ122772 | GenBank |
| 39 | K20-02 | B-3 | Gyeongsang | 2020.06 | PQ122740 | PQ122773 | GenBank |
| 40 | K20-03 | B-2 | Gyeongsang | 2020.07 | PQ122741 | PQ122774 | GenBank |
| 41 | K20-04 | B-2 | Gyeongsang | 2020.08 | PQ122742 | PQ122775 | GenBank |
| 42 | K20-05 | B-1 | Gyeonggi | 2020.09 | N/A | PQ122776 | GenBank |
| 43 | K20-06 | B-3 | Gyeongsang | 2020.09 | N/A | PQ122777 | GenBank |
| 44 | K20-07 | B-2 | Gyeongsang | 2020.09 | PQ122743 | PQ122778 | GenBank |
| 45 | K20-08 | Reassorted | Seoul | 2020.09 | PQ122744 | PQ122779 | GenBank |
| 46 | K20-09 | B-1 | Gyeonggi | 2020.09 | PQ122745 | PQ122780 | GenBank |
| 47 | K20-10 | A | Seoul | 2020.10 | PQ122746 | PQ122781 | GenBank |
| 48 | K21-01 | B-1 | Gyeonggi | 2021.06 | N/A | PQ122782 | GenBank |
| 49 | K21-02 | B-2 | Gangwon | 2021.09 | PQ122747 | PQ122783 | GenBank |
| 50 | K22-01 | E | Chungcheong | 2022.05 | PQ122748 | PQ122784 | GenBank |
| 51 | K22-02 | B-2 | Gyeongsang | 2022.06 | PQ122749 | PQ122785 | GenBank |

N/A: Genotyping was not available due to the low viral load, which was insufficient for sequence analysis.

**Supplementary Figure 1. Classification of participants based on previous study inclusion and feasibility for genotyping.**


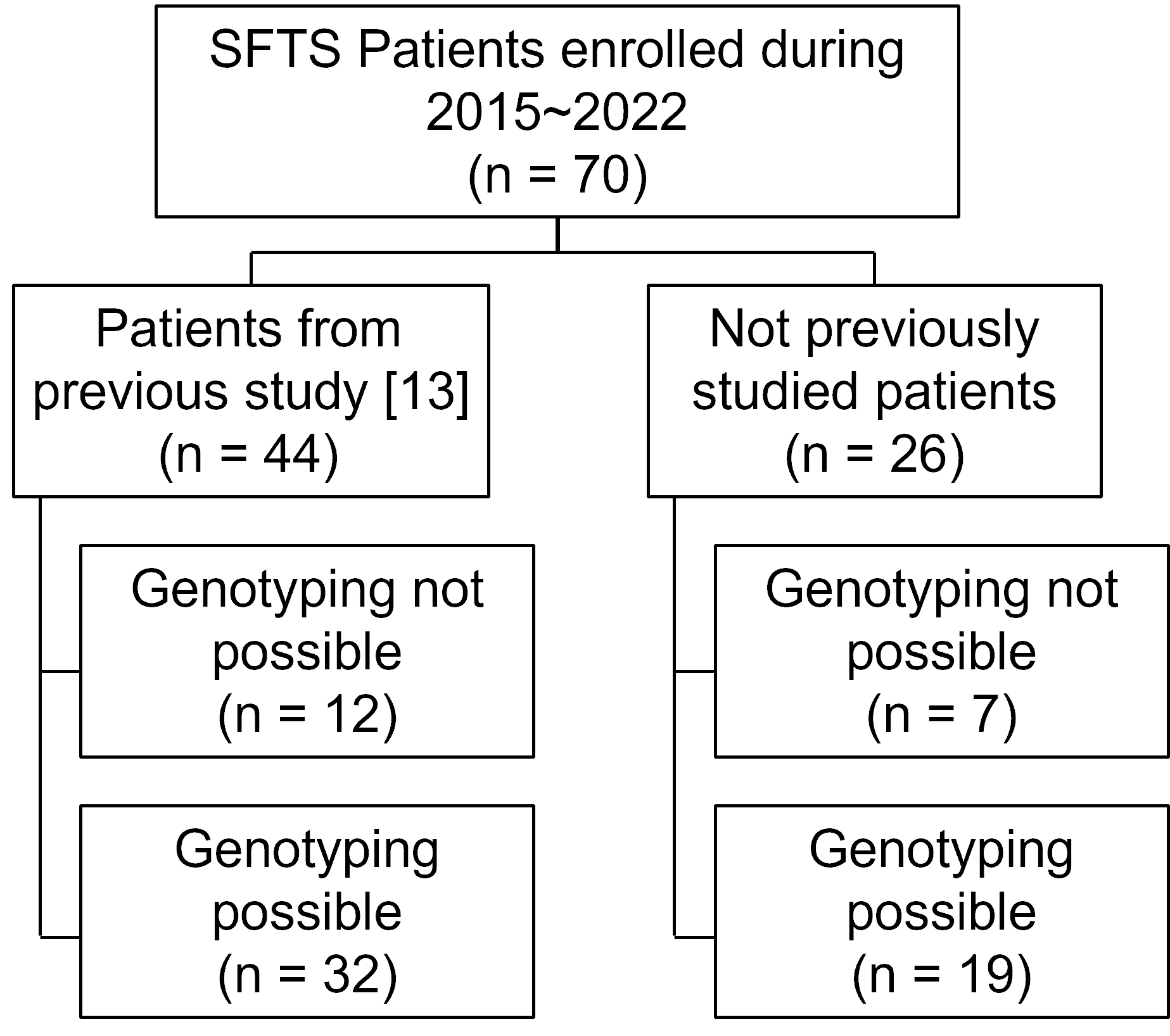

Supplement: ofae508_Supplementary_Data [file ofae508_supplementary_data.doc]
